# Pilomatrixoma of the Arm: A Case Report and Review of Literature

**DOI:** 10.7759/cureus.45768

**Published:** 2023-09-22

**Authors:** Nimah A Rabai, Arqam Alrababah, Saleh A Ba-shammakh, Ayat Aloqaily, Siwar W Saleh, Mahmoud M Al-Oqaily

**Affiliations:** 1 Department of General Surgery, Princess Basma Teaching Hospital, Irbid, JOR; 2 Department of General Surgery, Islamic Hospital, Amman, JOR; 3 Department of Pathology and Microbiology, Jordan University of Science and Technology, Irbid, JOR

**Keywords:** case report, basaloid cells, ghost cells, epithelioma, pilomatrixoma

## Abstract

Pilomatrixoma (PMX), also known as calcifying epithelioma of Malherbe, is a rare benign neoplasm that arises from the hair matrix cells, commonly in the head, neck, and upper trunk regions, infrequently affecting upper and lower extremities. It has to two peaks of presentation: under 20 years of age or between 50 and 65 years of age, slightly more common in females. The neoplasm exhibits diverse clinical manifestations and is frequently subject to misdiagnosis with alternative dermatological diseases. We present an atypical case of PMX affecting the upper extremity of a 62-year-old female patient. Surgical removal of the affected tissue under local anesthesia was performed, and subsequent histopathological analysis confirmed the presence of PMX. Based on the literature search we performed, we found out that this pathology is underreported in Jordan, with only one study published describing this tumor in the maxillofacial region. Physicians should be aware of this condition and its different presentations to include it in the differential diagnosis of suspected cases to provide the appropriate management and follow-up.

## Introduction

Pilomatrixoma (PMX) is a benign growth primarily observed in the skin, emerging chiefly from the matrix cells of hair follicles situated in the dermal or subcutaneous layers. First identified and recorded by Malherbe and Chenantais in the late 19th century, early interpretations suggested it to be a calcifying epithelioma associated with the sebaceous glands [1]. The understanding of the true origin of this tumor was later refined by Forbis and Helwig in 1961, where they pinpointed the hair follicle's cortex as the originating point, which led to the introduction of the term pilomatrixoma, better illustrating its genesis [2-3].

The occurrence of this neoplasm is significantly evident in two specific age groups: individuals aged between 0 and 20 years and a less prominent occurrence in the age group of 50 to 65 years [4]. It typically presents itself around the average age of 4.5 years, with a large fraction, about 90%, occurring in children aged under 10 years [5]. PMX mostly appears as a single, hard, stone-like mass, varying in size from 0.3 to over 3 cm, predominantly appearing on the face, neck, or upper trunk, particularly in children and younger adults [6-8]. These tumors generally have clear boundaries, move easily over the subcutaneous layer, and might exhibit a reddish or bluish coloration on the skin surface [5-6].

Diagnosing this tumor can be challenging due to its uncommon clinical features like multiple lesions or locations deep within the skin [9-10]. Identifying this condition predominantly relies on histological examinations, which unveil distinct lesions originating from the dermis and advancing into the adipose tissue beneath the skin. It's important to note that these lesions are typified by clusters of epithelial cells in conjunction with basophilic cells and, at times, ghost cells. Additionally, these examinations may show the presence of foreign body giant cells and calcifications [11].

A significant study done by Moehlenbeck in 1973 reviewed 140,000 samples of skin tumors and highlighted the uncommon nature of PMX, which accounted for only 0.12% of all skin tumor specimens analyzed [12]. A recent study has further quantified this rarity. It is estimated that PMX appears in roughly 0.1% of skin tumor instances, underscoring its rarity in the medical community [13].

Given its benign characteristics and infrequent occurrence, medical professionals need to have a deep understanding of PMX to ensure precise diagnosis and effective management, thus avoiding misidentifying it as other benign or malignant skin lesions [9].

In the Jordanian community, PMX remains notably rare and understudied, with only one documented study examining its clinicopathologic characteristics in the maxillofacial region [14]. There is a significant gap in the existing research regarding its incidence or prevalence in this demographic. This dearth of data highlights the urgent need for more research to enhance understanding and improve diagnostic and management approaches for PMX in Jordan. Conducting thorough clinical examinations can potentially augment diagnostic precision. In this report, we describe a rare case of PMX found on the left arm.

## Case presentation

A 62-year-old female patient, with a known case of controlled hypertension, presented to the general surgery clinic complaining of a small left arm mass for over 30 years, with a recent increase in pain, but no change in the size or color of the overlying skin. There is no history of trauma. No other masses were present. The patient has a negative family history of benign and malignant tumors.

On examination, a nodule was seen on the left arm posteriorly, approximately 3 cm proximal to the elbow joint, with no overlying skin discoloration or scar. The mass measured approximately 2.0 cm × 1.0 cm × 5 cm and was superficial, stony-hard in consistency, mildly tender on palpation, and slightly mobile with limited fixation to underlying tissue but not to overlying skin.

**Figure 1:**
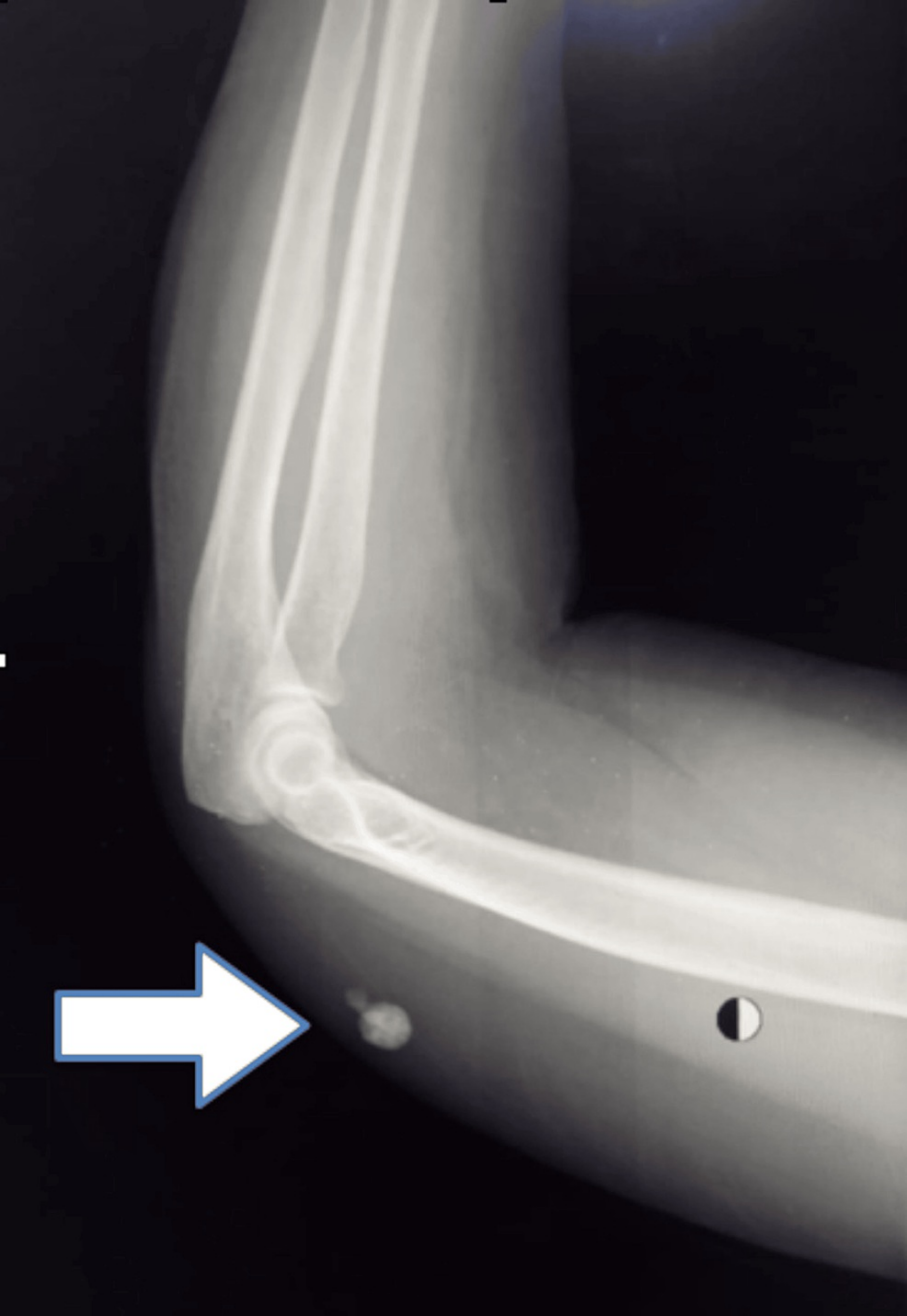
X-ray of the elbow shows a hyperdense lesion with variable calcification (arrow).

A left-arm X-ray was requested, and it showed a hyperdense oval lesion, measuring about 2.0 cm × 1.0 cm, approximately 3 cm above the elbow joint with variable calcification (Figure 1).

**Figure 2:**
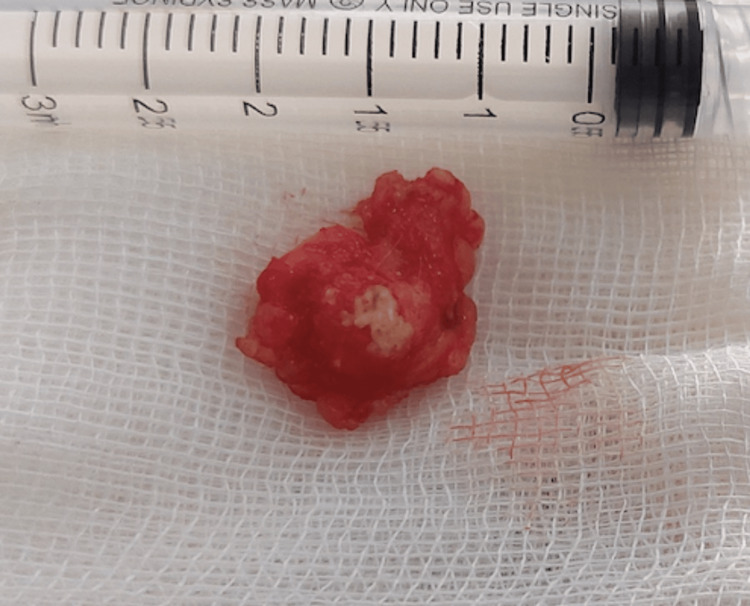
Gross appearance of the mass after excision.

The radiology and orthopedic physicians were consulted, and the impression was of a calcified lesion, possibly a suspected foreign body that could be due to unrecalled trauma. Other possible diagnoses included sebaceous cyst, dermoid cyst, adenopathy, or synovial sarcoma. As the patient had no medical insurance, she could not afford further investigations and requested the mass to be removed. So, based on the clinical impression of a benign pathology and respecting the patient's preference, we scheduled her for an excisional biopsy under local anesthesia. During the dissection of the mass, the mass felt bony hard when grasped by instruments, it was excised with some adherent surrounding tissue (Figure 2) and sent for histopathological examination.

**Figure 3:**
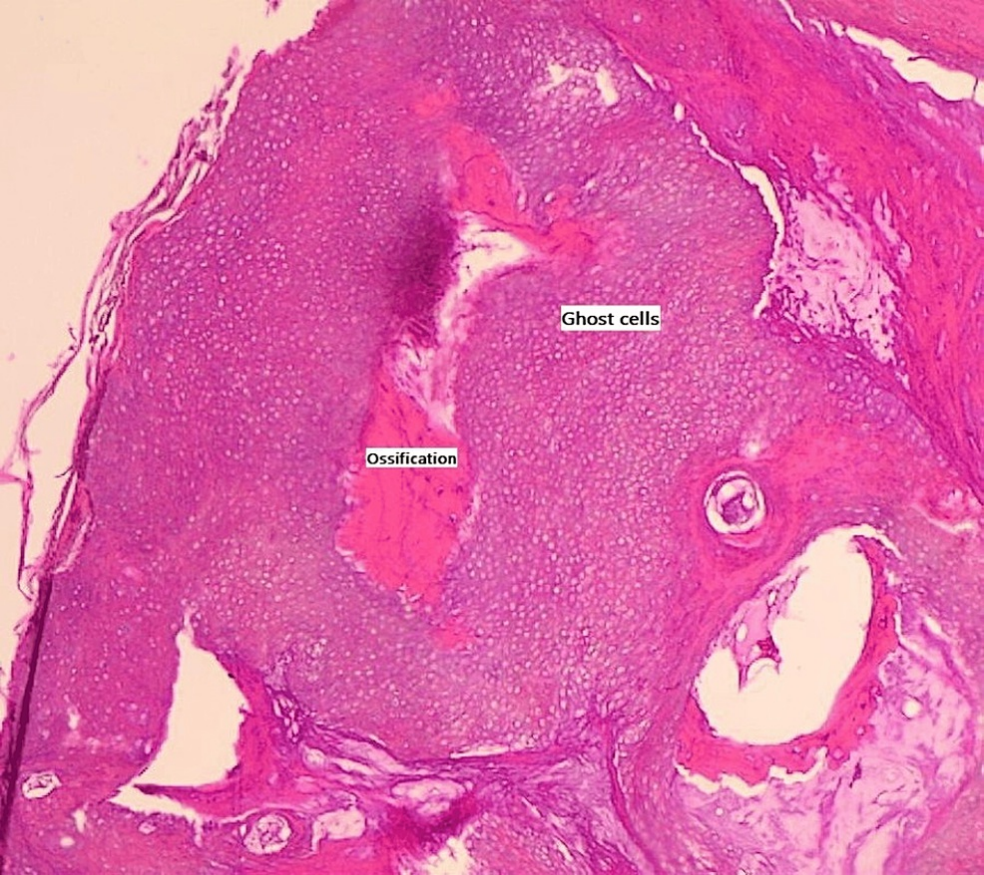
Microscopic examination of the nodule with H&E stain shows a nest of anucleated ghost cells with prominent intervening ossification and calcification. Magnification 10×. H&E, hematoxylin and eosin

Grossly, the mass measured 2.0 cm × 1.0 cm × 0.5 cm and was yellowish-white in color. Microscopically, it showed nests of ghost cells along with extensive calcification, ossification, foreign body reaction, and nests of vague basaloid cells, with no evidence of dysplasia or malignancy (Figure 3). These findings were consistent with PMX. The patient was asymptomatic and in a satisfactory condition following the surgery.

## Discussion

PMX is a noncancerous neoplasm of the skin that originates from the hair follicle matrix; it is most frequently found on the head, neck, and upper trunk but infrequently affects the upper and lower extremities [13]. With a ratio of 5:4, there is a slight female predominance [11]. Although PMX typically develops slowly over months or years, it can sometimes proliferate, similar to keratoacanthoma [5]. Two peaks of clinical presentation were noted in a clinical assessment of 209 PMXs: one significant peak occurred between 0 and 20 years of age, and the other, a less critical peak, occurred between 50 and 65 years of age [3]. Clinically, PMX manifests as a well-defined deep subcutaneous mass that is hard, stone-like, slowly developing, and may either be mobile or attached to the deep cutaneous tissues [11]. The superficial PMX may exhibit various characteristics such as atrophy, ulceration, bullous formation, telangiectasia, or keratosis, which can be mistaken for spindle cell carcinomas. Additionally, the overlying skin may appear normal or display a reddish or bluish tint, also known as the *tent sign* [14,15]. Its usual size ranges between 1 and 3 cm; larger masses <5 cm are described as giant PMX [16,17].

PMX is believed to be generated by a disruption in the life cycle of hair follicles brought on by trauma, inflammation, and radiation, according to several theories that have been reported [18,19]. In addition, there are associations between PMX and B-catenin gene mutations in the Wnt pathway [20]. The presentation of PMX is typically solitary, as observed in the current case. However, multiple lesions have been reported to be associated with various syndromes, including myotonic dystrophy, Familial Adenomatous Polyposis (FAP)-related syndromes, Turner syndrome, and Rubinstein-Taybi syndrome [8].

Physical examination, imaging, and cytology can all be used to establish a definitive diagnosis, yet frequent misdiagnoses remain. This might be attributed to the vast diversity of its clinical features [21] and doctors' possible lack of awareness of this tumor [22]. Dermoid cysts, foreign body granulomas, adenopathies, sebaceous cysts, hemangiomas, or malignant soft tissue tumors like synovial sarcoma are conditions linked to PMX misdiagnosis [23]. Our case was first misdiagnosed as a possible foreign body. However, no history of trauma was recalled by the patient, and the impression was based on examination, imaging, and, most importantly, related to the lack of knowledge regarding this condition.

The utilization of diagnostic imaging techniques, such as ultrasonography (US), computed tomography (CT), and magnetic resonance imaging (MRI), has been documented in rare cases. These imaging modalities have proven beneficial when the tumor is located in atypical sites, such as the parotid or breast [24]. Although ultrasound is more accurate than CT and MRI in diagnosing subcutaneous masses, PMX is still challenging to definitively diagnose using ultrasound [5]. Solivetti et al. [25] described five US patterns to differentiate PMX from other subcutaneous tumors.

A punch or excisional biopsy is the preferred diagnostic method for most cutaneous diseases in cytology. The fine-needle aspiration (FNA) technique is a fast and dependable method for procuring a preoperative diagnosis for specific cutaneous lesions [26,27]. Certain characteristics of PMX's cytological appearance on FNA are susceptible to misinterpretation for various skin conditions and malignancies [28]. Based on histology, PMX is characterized by the presence of two main types of cells: basophilic (basaloid) cells, which bear a resemblance to the matrix cells found in hair follicles, and shadow (ghost) cells, which are so named due to the central shadow that remains after the nucleus has disappeared [26,29].

The histological examination of the affected tissue reveals basaloid cells arranged in either grouped or separated patterns by pink fibrillary material. Additionally, squamous, ghost, and multinucleated giant cell co-occurrence is observed. These histological features indicate the possibility of PMX diagnosis [26]. According to the histological characteristics, Kaddu et al. [30] suggested that PMX has four morphological stages that proceed from an infundibular matrix cyst to a calcified cutaneous nodule. Early, fully developed, early, and late regressive stages are chronological. Our late regressive PMX had nests of ghost cells with significant calcification, ossification, and ambiguous basaloid cells, but no dysplasia or cancer.

The likelihood of distant metastasis and malignant transformation into pilomatrix cancer is quite low [31]. In most cases, wide surgical excision is still the most appropriate course of action, considering that incomplete surgical excision may result in postoperative recurrence [2]. Our patient underwent local excision of the mass with the adherent surrounding tissue, and it was excised entirely based on histopathological examination with no recurrence over one year of follow-up.

## Conclusions

PMX is an infrequent presentation in general surgery clinics. Upon searching published literature, we found this condition to be underreported in Jordan, which reflects the lack of knowledge and awareness of this pathology. Physicians should be aware of this neoplasm and its possible transformation into malignancy, including it in the differential diagnosis of suspected cases, and ensuring that appropriate treatment and follow-up are provided.
